# Bridging the Mechanical and the Human Mind: Spontaneous Mimicry of a Physically Present Android

**DOI:** 10.1371/journal.pone.0099934

**Published:** 2014-07-18

**Authors:** Galit Hofree, Paul Ruvolo, Marian Stewart Bartlett, Piotr Winkielman

**Affiliations:** 1 Department of Psychology, University of California San Diego, San Diego, California, United States of America; 2 Olin College of Engineering, Needham, Massachusetts, United States of America; 3 Institute for Neural Computation, University of California San Diego, San Diego, California, United States of America; 4 Emotient Inc., San Diego, California, United States of America; University of Perugia, Italy

## Abstract

The spontaneous mimicry of others' emotional facial expressions constitutes a rudimentary form of empathy and facilitates social understanding. Here, we show that human participants spontaneously match facial expressions of an android physically present in the room with them. This mimicry occurs even though these participants find the android unsettling and are fully aware that it lacks intentionality. Interestingly, a video of that same android elicits weaker mimicry reactions, occurring only in participants who find the android “humanlike.” These findings suggest that spontaneous mimicry depends on the salience of humanlike features highlighted by face-to-face contact, emphasizing the role of presence in human-robot interaction. Further, the findings suggest that mimicry of androids can dissociate from knowledge of artificiality and experienced emotional unease. These findings have implications for theoretical debates about the mechanisms of imitation. They also inform creation of future robots that effectively build rapport and engagement with their human users.

## Introduction

A key task for psychology and related disciplines is to identify mechanisms that allow humans to understand others, quickly respond to them, and coordinate mutual actions. One mechanism supporting these capacities is imitation [1], [2]. The core form of imitation is spontaneous mimicry, where merely observing another individual's behavior elicits a corresponding behavior in the observer without being instructed to do so [3]. The most basic form of such mimicry is the mirroring of emotional facial expressions—a phylogenetically ancient form of intra-species communication that occurs in humans and in some other primates [4]. As shown by a large body of research, the mere viewing of expressions can spontaneously and rapidly activate congruent facial muscles such that perceivers, for example, smile in response to a smile and frown in response to a frown [5], [6]. Such behavior can, under some conditions, support recognition of emotional states (e.g., [7], [8]). In general, spontaneous mimicry promotes empathy, rapport, contagion, and social coordination [9], [10] via a variety of psychological and neural mechanisms [9], [11], [12].

Researchers interested in imitation have long utilized robots as research tools. Such research has shown, for example, that voluntarily mimicking robots leads to more positive interactions [13], [14] and that the speed of rudimentary human motor responses is influenced by compatibility with a movement of a simple robotic hand [18]. Critically, new discoveries about the underlying mechanisms, as well as social consequences of imitation and mimicry, are now made possible by developments of so called “hyper-realistic androids”—robots that possess a humanlike face and produce believable emotional expressions. As elaborated shortly, evidence of spontaneous human mimicry of such androids would have important implications for major theories of mimicry. Moreover, such evidence would be important for informing theoretical questions of robotics, such as synchronization between robotic and human agents, the role of emotions in human-robot interaction, and the role of androids' physical presence in cognitive and emotional responses. Answers to these questions are also practically important since such agents are currently being developed, at great cost, for households, education, customer service, and care for the elderly and disabled. Further, the lifelike appearance of these robots is presumed to enhance human welfare [15] in situations where the natural social interaction between robots and humans is a crucial component to success in these efforts [16]. Interestingly, for practical purposes androids often resemble well-known humans (e.g., Repliee, various actroids, and Hanson's Einstein). Evidence of spontaneous mimicry of such realistic androids could facilitate creation of agents that effectively build social rapport and create a new level of engagement with their human users, using a naturalistic non-verbal, high-bandwidth robot-human communication channel.

### Theoretical Accounts that Predict Mimicry of Androids

As mentioned, our main focus here is on how hyper-realistic androids allow insights into the mechanisms of spontaneous mimicry. Note that different theoretical accounts make different predictions regarding the emergence and the exact nature of android-human mimicry. Later we will discuss accounts that doubt the very possibility of android mimicry. But, first, let us consider some theoretical accounts that predict robust human mimicry of such agents. Note that some of these accounts are generally concerned with motor mimicry, whereas others focus more on emotional processes.

One general ‘motor’ account, termed associative sequence-learning (ASL), assumes that mimicry reflects relatively low-level, automatic operations involved in sensorimotor mapping [17]. According to ASL, facial mimicry represents a subclass of general stimulus-response compatibility effects, in which observation of a task-irrelevant action facilitates a similar action in the perceiver, by virtue of repeated previous pairings of perception and motor execution. In short, as long as the novel stimulus is reasonably similar to stimuli that were previously associated with specific motor actions, it should trigger these same actions. The face of a hyper-realistic humanoid robot is certainly such a stimulus, and indeed the prediction of robust mimicry is consistent with early work reporting imitation of robotic hands [18].

Another account that predicts robust mimicry of an android's expression assumes that mimicry reflects involvement of a sensorimotor loop aimed at facilitating stimulus recognition. Specifically, facial mimicry represents an automatic embodied simulation that provides motor feedback to the perception process, and thereby helps distinguish subtle differences in the observed emotional expression [19]. According to this account, as long as people are trying to recognize a face, regardless of whether this faces is actually human or robotic, they should use such an embodied simulation strategy.

The above accounts (ASL and automatic embodiment) focus on general processes, and assume that the process underlying facial mimicry is essentially motoric in nature. Other accounts that predict robust mimicry of an android's expressions focus on emotional processes. One such account, sometimes termed the “affect-matching account”, assumes that seeing facial expressions automatically triggers corresponding affective responses in the perceiver [20]. For example, seeing a smile induces positive affect, which then triggers a smiling expression. If an android produces an expression that is highly similar to an actual human smile, it may elicit positive affect in the human perceiver, and thus trigger smiling. It is worth noting that while the motor and affect accounts agree that an android can trigger facial mimicry, the two theories make slightly different predictions regarding the exact nature of corresponding muscle responses. The motor accounts assume that the process is strictly imitative and thus the produced expression should closely mirror the shape and dynamics of the observed expression. The affect-matching account suggests that the shape and dynamic of a facial response reflects the internal nature of the triggered emotional process. In this sense, “mimicry” is a slightly misleading term, though often used, for such “matched” responses, as the similarity between perception and production comes from a match in affective state rather than motoric features. Accordingly, the affect-matching account predicts that facial “mimicry” responses can sometime disassociate from the perceived expression, as when observation of a fearful expression triggers negative affect expressed by facial frowning – a feature absent in the eliciting “fearful” stimulus [21]. We will return to this issue in the discussion, and only note that we use the term mimicry technically, without committing to a particular interpretation. For now, the important point is that all accounts discussed so far predict that humans will mimic expressions of an android, especially a state-of-the-art android that closely resembles a human being.

### Perception of Human-Likeness in Androids

But what does it mean for an android to “resemble a human being?” Most views agree that some perception of an android's “human-likeness” plays a necessary role. However, they disagree on what aspects of being “humanlike” are essential. Some views, which we will explicate later, emphasize the role of *psychological similarity*, where the critical variable is the perception of the ability to have “intentional states, such as beliefs, desires, and emotions” [22]. Other views emphasize the role of physical similarity, where the critical variable is the perception of overlap in external appearance between humans and androids. This sense is closest to the Latin meaning of anthropomorphism, which is about human form or shape [18], and might also be informed by perception of human motion [23]. The possible importance of anthropomorphic form or motion is consistent with recent findings from the human-robot interaction (HRI) field demonstrating that people respond facially to non-human agents that are physically similar to humans. For example, people react with similar facial expressions to a video of a robotic face when asked to identify the emotion it is displaying [24]. Another study reports that people respond to a head gesture mimicking chimpanzee robot with similar gestures [14]. Avatars—virtual human characters, can also elicit facial mimicry in observers [25]–[27]. In short, if anthropomorphism is the key to unlocking mimicry and relatively superficial appearance-based humanlike features can trigger automatic mimicry, then an ultra-realistic android should be even more likely to induce mimicry reactions.

Critically, the perception of human-likeness may involve more than perception of psychological and/or physical similarity. Recent work emphasizes the role of the actual physical “presence” of the robot. In fact, some research suggests that merely sharing the same physical space with an android influences how humanlike it is perceived to be [28]. This is important because it suggests that actual presence may enhance the emergence of mimicry. More generally, the role of presence highlights the theoretical and practical value of investigating the impact of actual, physically-present robots. While avatars on a computer screen can be quite realistic, they hardly approach the details of an android. After all, these androids often have actual hair, human-like skin, clear eyes, and other salient features that are normally encountered only in real, living humans. These androids are also collocated in the human's personal space — they are tangible, within human reach, and have the human within their reach. In addition, while people are very familiar with robots and animated on-screen avatars (since TV and movies provide an alternative reality in which robots and avatars are plausible), they are unfamiliar with physically present androids. The novelty of such an experience, as well as the more concrete representations of the android brought about through spatial proximity [29], might further highlight these humanlike features. Nonetheless, the experience of presence appears to be a complex phenomenon, and it is yet unclear how visual realism contributes to it [30]. Interactions with real robots might therefore lead to qualitatively different reactions. So, while the earlier work on nonhuman agents leaves open whether subjects will imitate a realistic android, it is certainly suggestive of this possibility.

### Theoretical Accounts Predicting No Mimicry of Androids

As mentioned earlier, there are some alternative theoretical perspectives that speak against the possibility of human mimicry of androids. One prominent view emphasizes that mimicry is a tool for understanding the observed agent's emotions and intentions from “within,” via perceiver's simulation of the agent's mental states (for a review, see [31]). Under a strong interpretation of this view, mimicry will occur only if the perceiver believes that the observed agent actually possesses mental states (otherwise, there is nothing to find out from “within”). In fact, there is some evidence that “psychological anthropomorphism,” (or the belief that that an agent shares human-like intentions and emotion) predicts many emotional responses to other agents [32], [33]. According to that view, people should not mimic an android, if they indeed believe that it lacks intentionality and emotion.

Another view that predicts an absence of android mimicry emphasizes the role of emotional unease humans might experience when confronted with an android – an eerily human-like, yet clearly artificial being [34]–[36]. This prediction is based on evidence that a human's negative attitudes towards a model generally reduce or even reverse mimicry [19], [25], [37], [38]. Note that emotional unease might be exacerbated when interacting with an android face-to-face, due to a salient mismatch between its humanlike appearance and robotic features (such as mechanical sounds or jerky un-humanlike movements). In short, negative emotions should lead to disengagement and discourage relating to the android in the form of mimicry.

Current Research

Our research examined the issues discussed above in two studies that tested mimicry of a hyper realistic android. Specifically, we explored whether people will spontaneously mimic such an android, and whether the strength of mimicry depends on the android's actual physical vs. virtual presence (via video). In addition, we tested how mimicry depends on (i) the perceptions of human-likeness and intentionality, and (ii) the feelings of emotional unease about the android. In both studies, we employed Hanson's Einstein, a state-of-the-art android programmed to perform realistic human expressions (see sample video of Einstein: http://pages.ucsd.edu/~pwinkielman/einstein_happy_angry.mov). In Study 1, we compared participants' mimicry reactions in response to videos of the android and to a matched human control making the same expressions. In Study 2, we examined participants' reaction to a physically present android—where humans sit face-to-face with the actual robotic agent. In addition, we had participants assess the android on human-likeness, emotional comfort, as well as intentionality.

Our predictions were as follows. Based on theoretical views emphasizing the automaticity of mimicry-related processes, we predicted that the android would elicit spontaneous human mimicry. Furthermore, such mimicry should occur even when participants do not perceive the android as having intentionality, and even when participants experience psychological discomfort with the agent. Based on the idea that mimicry requires a sense of similarity and relatedness between the self and the other [16], [18], we also predicted that mimicry should be magnified by perceptions of human-likeness as well as the physical presence of the robot.

## Study 1

### Methods

#### Robot design

**Figure 1:**
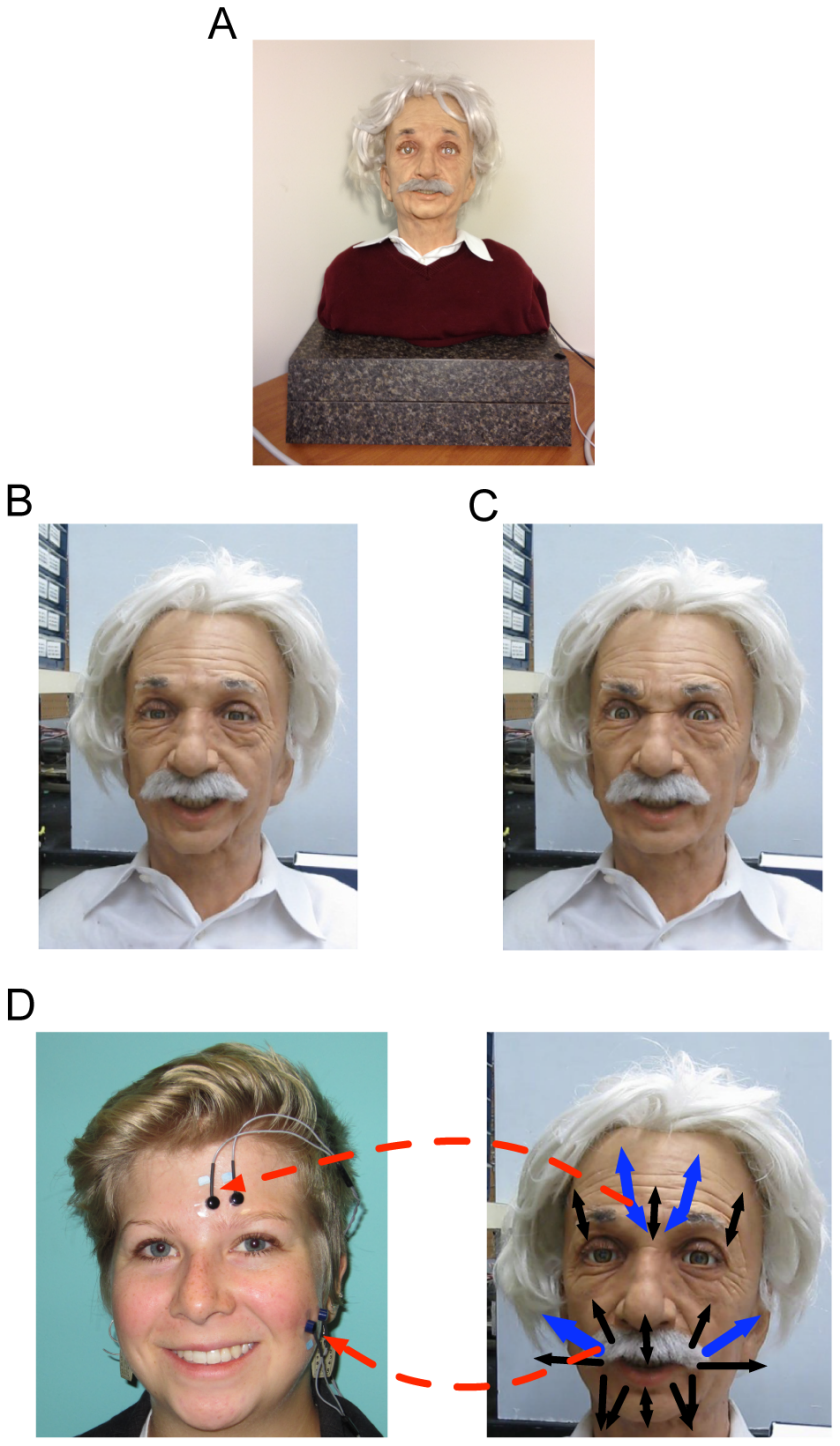
Linking android and human facial expressions – servos vs. muscles. (**A**) Einstein the robot, as the participants saw him. Robot expressions of (**B**) happy and (**C**) angry. (**D**) Relationships were investigated between the actuators shown in blue, and facial muscle activity in the corrugator and zygomaticus, as indicated by the red arrows. The subject in the photograph has given written informed consent, as outlined in the PLOS consent form, to publication of their photograph.

Both studies used an android manufactured by Hanson Robotics. This is one of the most advanced androids available to study human-robot interactions, and its facial behavior has been programmed according to the highest standards in the field [39]. Its face, shown in Figure 1A, is made of skin-like materials and is actuated by 31 servos (motors) programmed to perform facial movements that closely match human expressions, such as happiness and anger (Figure 1B/C, see also File S1). As with other hyper-realistic androids, its appearance is personalized by resemblance to a well-known human — in this case, Albert Einstein.

#### Videos

We created 6 second videos of the robot and an aged-matched human control making happy and angry expressions. To make the videos fully comparable between agents and emotions, both in terms of the temporal dynamics and the intensity of the expression, we made two versions of each expression: (i) natural and (ii) intensity-matched. The natural videos were created by asking the control human to watch the android's expressions and mimic them as closely as possible. This matches the videos on subjective perceptions of similarity. The intensity-matched videos were edited in such a way that the peak intensity of the expression matched between the android and the human control (as judged by a FACS expert). The onset, peak time, and offset of the expressions were the same, thus formally standardizing our videos both across time and intensity.

#### Pretest - Anthropomorphic Ratings of Android and Control Videos

We first tested whether people attribute mental states to the android, which according to some views discussed earlier, is essential for mimicry. We ran this pretest separately so as not to interfere with the measurement of spontaneous mimicry reactions. In this pretest, we used the widely used Individual Differences in Anthropomorphism Questionnaire, or IDAQ [40]. It includes questions regarding the mental states (e.g., intention, free will, and emotion) of technological devices, as well as non-human animals, and natural entities. In addition, we constructed a modified IDAQ with the same questions regarding the android and the human control. During the pretest study, 392 separate UCSD participants (300 female) first filled out the standard IDAQ questionnaire. Next, participants watched happy and angry (matched-intensity) videos of both the android and the control (counterbalanced) and then filled out a modified IDAQ about the android and the human control.

The results of this pretest show that participants rated the human control as overall higher in intentionality than the android (M_android_ = 3.55, M_control_ = 7.39, *t*(382) = 20.17, *p*<0.0001, *d* = 2.06). In fact, participants perceived the android as significantly less capable than the human control of mental states such as free will, consciousness, and emotion (all ps<0.0001), rating him near the low end of the scale for each of these states. For comparison purposes, the android was rated as possessing more free will than a car, but less than a fish (M_android_ = 3.19, M_car_ = 0.72, M_fish_ = 5.7). Android consciousness was rated above that of an average robot, but less than a reptile (M_android_ = 3.27, M_robot_ = 1.45, M_reptile_ = 6.43). The android was rated as experiencing more emotions than a television set, but less than a cheetah (M_android_ = 4.25, M_TV_ = 1.21, M_cheetah_ = 7.14). The human control was rated above all of the examples used in the IDAQ, as expected. Interestingly, individuals' responses to IDAQ questions regarding technological devices predicted the difference between the human and android ratings. Specifically, those who considered technology as being more anthropomorphic also rated the android and the human control more similarly on anthropomorphism (*b* = −0.59, *t*(380) = −5.39, *p*<0.0001, *d* = 0.55). Gender of the participants had no effect on these ratings. It appears that individuals' general tendency to anthropomorphize technology was associated with attributing more internal humanlike properties to the android (e.g., intentions and emotions). Overall, the critical point here is that participants rate the android low in intentionality.

#### Participants in the main experiment

Forty-eight undergraduates from University of California, San Diego (UCSD) participated in this experiment (30 female and 18 male). The research protocol was approved by UCSD Institutional Review Board and all participants provided written informed consent. Twelve subjects were excluded from EMG analyses due to corrupt data, 2 were excluded from the experiment due to their familiarity with the android or human control, and another 5 were excluded from the experiment because they guessed that we were measuring facial expressions or mimicry specifically.

#### Ratings during the experiment

We were especially interested in how mimicry depends on specific kinds of perception: (i) the sense of “humanlike” quality of the android and (ii) the sense of comfort with the android. Thus, we had participants make such ratings, for both the android and human (intensity-matched) videos, before and after the mimicry phase. In addition, after the experiment, participants also rated their arousal, negative feelings, and positive feelings. The android was also specifically rated on “creepiness” (specifically, we asked how creepy/scary/weird subjects found the robot). Answers to these questions were measured on 9-point Likert scales, ranging from 1 (not at all), through 5 (moderately), to 9 (very).

#### Procedure

As just mentioned, participants first rated both agents (robot and control) on comfort and humanlike attributes (presentation and ratings were counterbalanced). Then, they proceeded to the mimicry part of the study, which was modeled after a classic paradigm [5], [6]. Participants viewed randomly interspersed videos of the android and the control, each displaying happy and angry expressions. As in previous research, there were two phases. In the first, spontaneous phase, participants were instructed to simply observe the videos, without receiving any instructions or encouragement to mimic. In the second, intentional phase, participants were told to “try and make the same face as the object in the video.” This intentional condition was included to ensure that the human and androids' expressions are easily visible and principally imitable and that our EMG paradigm can accurately detect mimicry. The order of these two conditions was kept constant so as to avoid the introduction of an implicit expectation for mimicry behavior in the spontaneous phase.

In each condition, there were separate blocks of natural videos, along with matched-intensity expression videos, each with 40 trials. Following the mimicry tasks, participants again rated both agents on various attributes (see above). We gauged participants' mimicry behavior using facial electromyography (EMG), a technique that measures electrical changes in underlying muscle activity, thus allowing for fast and sensitive online assessment of participants' facial reactions to the android's expressions. Our methods follow the official and published standards for EMG recording, collection, analyses and data presentation [41], [42]. Following these standards, electrodes were placed over the cheek muscle (zygomaticus major) and the brow muscle (corrugator supercilii) (for more details, see File S1).

### Results

#### Pre-experiment ratings

As expected, participants found the android to be significantly less humanlike (*M_android_* = 4.32, *M_control_* = 7.42, *t*(37) = 8.89, *p*<0.0001, *d* = −1.44), and felt less comfortable viewing his videos than those of the control (marginally significant two-tailed t-test: *M_android_* = 4.11, *M_control_* = 4.68, *t*(37) = 1.83, *p* = 0.08, *d* = 0.29). There were no gender differences in these ratings.

#### Post-experiment ratings

Participants' responses were consistent with their pre-experiment ratings of the android and the control on comfort and humanlike attributes. The only exception was ratings of how humanlike they found the control; they rated the control as significantly more humanlike following the experiment (*M_pre-mimicry_* = 7.42, *M_post-mimicry_* = 8.05, *t*(37) = 2.27, *p* = 0.03, *d* = 0.38). In addition, they rated the android as significantly less arousing (*M_android_* = 1.95, *M_control_* = 2.58, *t*(37) = −2.77, *p* = 0.009, *d* = −0.47) and less positive (*M_android_* = 2.34, *M_control_* = 3.16, *t*(37) = −2.75, *p* = 0.009, *d* = −0.45), but not more negative (minimal gender differences were found, see File S1). Finally, they also rated the android as more than moderately creepy (t-test comparing mean to 5, which was our scale “moderate” midpoint): *M_creepy_* = 5.48, *t*(37) = 2.11, *p* = 0.04, *d* = 0.34). In summary, our participants find the android in the videos not very humanlike, especially when compared to the human control, and feel less comfortable viewing it, to the point of finding it rather “creepy”.

#### EMG data

Analyses focused on the following predictions. If humans mimic, then shortly after the appearance of the target's expression, they should show greater EMG activity in the brow area for anger and cheek area for happiness. Critically, such mimicry should occur in the spontaneous condition. As discussed earlier, spontaneous mimicry of the android should be modulated by individual ratings of human-likeness [17], [18]. The intentional condition should obviously produce strong mimicry, in response to both the android's and the human's expressions.

Specifically, we compared EMG activity between happy and angry trials, for both agents and both muscles. We expected mimicry-related EMG activity to be as follows: activity of the corrugator muscle should be elevated during angry trials, as compared to happy trials, while the reverse should occur for the zygomaticus muscle. Note that if mimicry closely matches stimulus characteristics, there should also be some zygomaticus activity during angry trials. This is because the android's angry expressions also contain a “grimace” involving its cheek (see sample video linked in the Introduction).

Data were analyzed by comparing happy and angry trials separately for each muscle using a repeated-measures MANOVA over 500 millisecond intervals of a 6 second trial (i.e., 12 time points). Because spontaneous mimicry reactions can occur rapidly [5], [20], we also conducted similar MANOVAs over 200 millisecond intervals of the first second of the trial (i.e., 5 timepoints). Time was included as a factor in all analyses to account for changes in EMG responses over the course of the trial. Gender effects were tested and are reported separately in File S1. Before the main analyses, we collapsed responses to the matched-intensity and natural videos, as preliminary analyses did not reveal any effect of these conditions. Next, we first present our analyses conducted on each agent (android, human) separately, and then discuss an omnibus ANOVA that includes agent type as a factor. In addition, we tested the role of humanlike perception of the android. To do so, we conducted mimicry analyses on participants who responded high or low on the human-like rating, using a median split. Finally, to test whether emotional unease decreases mimicry, we conducted similar analyses on participants who responded high or low on comfort ratings [25].

#### Spontaneous mimicry

**Figure 2:**
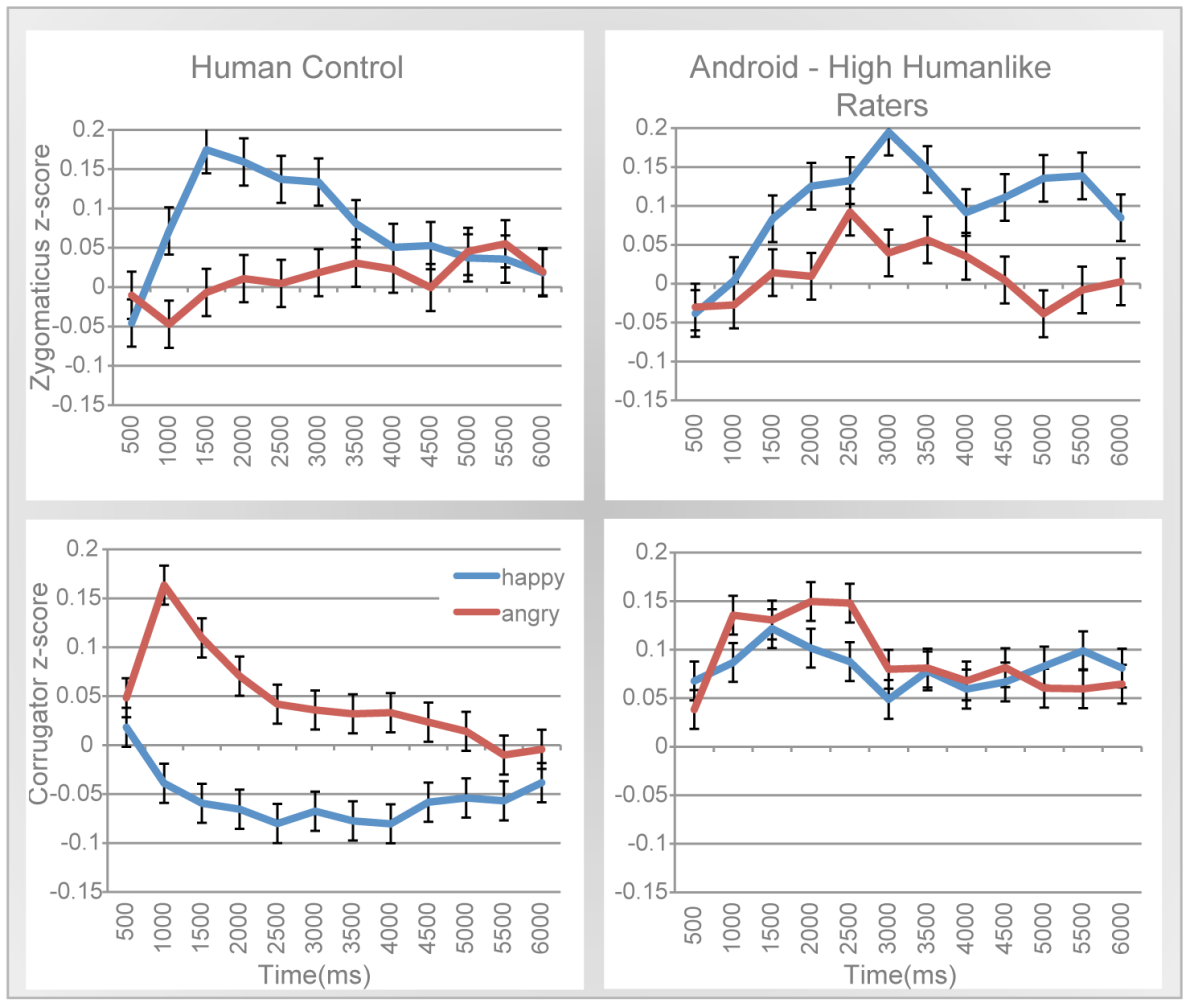
Spontaneous mimicry in both muscles for the human control and android videos, for those who rated the android high on humanlikeness. Zygomaticus activity (top) and corrugator activity (bottom) across 6 second trials in response to a human control (left), and in response to the android (right). Those who rated the android high on humanlikeness (right) showed mimicry reactions in the zygomaticus muscle (top right).

We first tested whether we could replicate the standard phenomenon of spontaneous mimicry in the human control condition. Figure 2 shows that participants clearly mimicked the human control, in both muscles. As shown in Figure 2 top left, zygomaticus activity during happy trials significantly increased from the beginning of the trial to peak at 1.5s, *t*(28) = 2.22, *p* = 0.03, *d* = 0.84. During angry trials, zygomaticus activity did not differ from baseline, as demonstrated by a non-significant contrast comparing activity across all 12 timepoints to zero, *t*(11) = 1.32, *p* = ns. Correspondingly, the zygomaticus demonstrates a significant Emotion × Time interaction, due to participants smiling more and earlier at happy expressions, than angry expressions, *F*(11,308) = 2.67, *p* = 0.003, *partial η* ^2^ = 0.09. Analyses of rapid response (200 ms intervals) demonstrate that this mimicry reaction begins within the first second (Emotion × Time interaction: *F*(4,112) = 3.37, *p* = 0.012, *partial η* ^2^ = 0.08).

The analysis of the corrugator muscle for the Human Control condition revealed a significant main effect of Emotion, such that participants frown more to angry than happy expressions, *F*(1,28) = 4.06, *p* = 0.05, *partial η* ^2^ = 0.13. As illustrated in Figure 2 bottom left, there is also a significant Emotion × Time interaction, where the corrugator is activated more in the beginning portion of the trial, peaking at 1s and diminishing by 3 s, *F*(11,308) = 2.61, *p* = 0.003, *partial η* ^2^ = 0.09. A MANOVA conducted over the 200 ms intervals of the first second of the trial reveals both a main effect of emotion (*F*(1,28) = 5.75, *p* = 0.023, *partial η* ^2^ = 0.17) and a significant Emotion × Time interaction (*F*(4,112) = 4.71, *p* = 0.002, *partial η* ^2^ = 0.14), demonstrating that corrugator associated mimicry also occurred very early into the trial. In summary, we observed robust spontaneous mimicry to human facial expressions.

**Figure 3:**
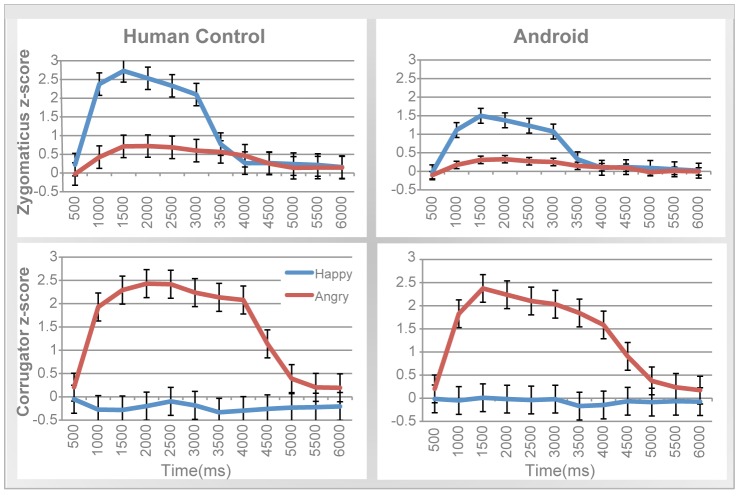
Intentional mimicry in both muscles for the android and human control. Zygomaticus activity (top) and corrugator activity (bottom) across 6 second trials in response to the human control's facial reactions (left), and in response to the android (right).

Next, we analyzed mimicry to the android. As mentioned earlier, we were especially interested in how perception of the android's similarity to humans might influence mimicry. For this reason, we also tested if mimicry depends on whether participants initially rated the android as low or high in human-likeness (median-split). Indeed, these two groups react to the android very differently. This is most evident in the activity of the zygomaticus muscle. A MANOVA including low/high human-likeness as a factor reveals a significant Human-Likeness × Emotion interaction, *F*(1,27) = 10.94, *p* = 0.003, *partial η* ^2^ = 0.29. This difference in reaction is already apparent within the first second of the trial (Human-Likeness × Emotion: *F*(1,27) = 9.3, *p* = 0.005, *partial η* ^2^ = 0.29). When examining just those participants who rated the robot highly on human-likeness, we found that they show significant signs of mimicry (main effect of Emotion: *F*(1,17) = 6.91, *p* = 0.02, *partial η* ^2^ = 0.29), smiling more to happy expressions than to angry expressions (Figure 3 top right). However, these reactions to the android are still weak compared to the human stimulus. This is formally demonstrated by an analysis that added Agent type to the above MANOVA conducted only on participants who rated the android high in human-likeness and revealed an Agent × Emotion × Time interaction, *F*(11,187) = 2.457, *p* = 0.007, *partial η* ^2^ = 0.13. A MANOVA with human-likeness included for corrugator activity did not yield any significant effect of Emotion. Interestingly, corrugator responses for both expressions went up from the baseline (contrast comparing activity across all 12 timepoints to zero: *happy*: *t*(11) = 16.69, *p*<0.0001, *d* = 10.06; *angry*: *t*(11) = 16.79, *p*<0.0001, *d* = 10.12). Similar analyses including a median split of comfort ratings revealed no significant interaction with mimicry reactions in either muscle.

Finally, we conducted a full repeated-measures MANOVA comparing happy and angry trials that also included Agent (android and human control) as a factor. This analysis revealed some overall differences between reactions to the robot and the human. The corrugator muscle shows a significant main effect of Agent, such that participants frown more overall to the human, *F*(1,28) = 9.37, *p* = 0.005, *partial η* ^2^ = 0.25. Most likely this reflects that participants only mimicked happy expressions of the android (and not angry expressions), while they mimicked both the angry and happy expressions of the human. There was also a significant Agent × Time interaction for the corrugator (*F*(11,308) = 2.5, *p* = 0.005, *partial η* ^2^ = 0.08) and a significant Agent × Emotion × Time interaction for the zygomaticus, *F*(11,308) = 2.35, *p* = 0.009, *partial η* ^2^ = 0.08. No effects of gender were found for the spontaneous condition for both muscles. Conducting similar MANOVAs over the first second of the trial demonstrated that differentiation in response to the agents begins very early. Similar to the full trial MANOVAs, a main effect of Agent was found in the corrugator (*F*(1,28) = 4.20, *p* = 0.05, *partial η* ^2^ = 0.13), where in the zygomaticus a significant Agent × Emotion interaction (*F*(1,28) = 10.94, *p* = 0.03, *partial η* ^2^ = 0.16) demonstrates that participants begin smiling early to the control, but not to the android. Overall, these results suggest that participants are quicker to react to the expressions of the human control, as compared to the android.

#### Intentional mimicry

Figure 3 shows intentional EMG activity across all time-points in a trial in both muscles (for both the android and the control). It is clear from this figure that both agents elicited robust mimicry. This was confirmed by statistical analyses of EMG activity for the android and the control separately.

Figure 3 top right illustrates the results for the android. On the zygomaticus muscle, there was strong, significant main effect of Emotion, *F*(1,28) = 34.36, *p*<0.001, *partial η* ^2^ = 0.55, as well as a significant Emotion × Time interaction, *F*(11,18) = 6.49, *p*<0.001, *partial η* ^2^ = 0.80. Participants are clearly smiling more to android's happy expressions than angry expressions. Interestingly, there is also some evidence that in this condition participants are also grimacing in response to angry expressions (linear contrast comparing to a zero baseline: *t*(11) = 8.04, *p*<0.0001, *d* = 2.41). Also, it appears that zygomaticus activity occurs earlier and stronger for happy expressions than for angry expressions. As illustrated by Figure 3 bottom right, the corrugator muscle also shows a significant main effect of Emotion, (*F*(1,28) = 126.42, *p*<0.001, *partial η* ^2^ = 0.82), along with an Emotion × Time interaction, *F*(11,18) = 16.83, *p*<0.001, *partial η* ^2^ = 0.91. As expected, participants frowned to the android's angry expressions but not its happy ones.

For the human control, we saw similar results, as shown in Figure 3 left top/bottom. That is, we observed a main effect of Emotion for both the corrugator and zygomaticus muscle (*corrugator*: *F*(1,28) = 170.67, *p*<0.001, *partial η* ^2^ = 0.86; *zygomaticus*: *F*(1,28) = 43.60, *p*<0.001, *partial η* ^2^ = 0.61), as well as significant Emotion × Time interaction for both muscles (*corrugator: F*(1,28) = 14.05, *p*<0.001, *partial η* ^2^ = 0.90; *zygomaticus*: *F*(11,18) = 10.52, *p*<0.001, *partial η* ^2^ = 0.87). These intentional mimicry reactions are initiated early in the trial, as can be seen in analyses conducted over the first second of the trial. We find both a main effect of Emotion and Emotion × Time interaction in both the zygomaticus and corrugator muscles, for both the android and the control (see File S1).

The preceding analyses make clear that intentional mimicry was robust to both the human and the android. Nonetheless, when including Agent into the MANOVA (as before), we again found evidence for a difference in reaction to the android and the human control. The zygomaticus showed a significant main effect of Agent (*F*(1,28) = 57.63, *p*<0.001, *partial η* ^2^ = 0.67), a significant Agent × Time interaction (*F*(11,18) = 9.21, *p* = 0.005, *partial η* ^2^ = 0.71), and a significant Agent × Emotion × Time interaction (*F*(11,18) = 2.7, *p* = 0.03, *partial η* ^2^ = 0.63), as well as the expected effect of Emotion (*F*(1,28) = 131.62, *p*<0.0001, *partial η* ^2^ = 0.67) and Emotion × Time interaction (*F*(11,308) = 41.35, *p*<0.0001, *partial η* ^2^ = 0.60). In short, while participants mimic both the android and the control, they are clearly reacting more to the expressions of the human control than to that of the android. More specifically, they smile more to the control's happy expressions and grimace more to the control's angry expressions. In terms of the corrugator, it showed the expected effects of Emotion (*F*(1,28) = 175.59, *p*<0.0001, *partial η* ^2^ = 0.86) and Emotion × time interaction, *F*(11,308) = 71.32, *p*<0.0001, *partial η* ^2^ = 0.72. More interestingly, the corrugator also demonstrated a significant Agent × Time interaction (*F*(11,18) = 5.62, *p* = 0.001, *partial η* ^2^ = 0.25) and a significant Agent × Emotion × Time interaction (*F*(11.308) = 2.98, *p* = 0.001, *partial η* ^2^ = 0.10). Figure 3 (bottom panels) illustrates that participants' frowning response to the human control's anger expression peaks for a longer duration than their frowning response to the android. Note also that participants show differentiated reactions to the two agents early in the trial in both muscles, as can be seen in analyses conducted over the first second of the trial (see File S1). It is also worth noting that some gender effects were found for both muscles in this condition (see File S1).

### Discussion

This study, in which participants watched videotaped facial expressions of an android and a control human, revealed several interesting findings. The analyses of ratings revealed that participants clearly find the android less positive than the human control, and even see him as more than moderately “creepy.” Moreover, they find the android less humanlike, and attribute less intentionality to him, compared to the human control. It is also worth highlighting again that in the pretest ratings, the android was rated as less conscious than a reptile, having less free will than a fish, and less emotion than a cheetah. In short, in terms of intentional states, our android was rated low.

Still, some participants did spontaneously mimic the android. Interestingly, those participants also rated the android high on human-likeness. The ratings of emotional discomfort, or intentionality, did not modify spontaneous android mimicry. As such, the results of Study 1 reveal that some aspect of “human-android” relationship plays a critical role in spontaneous mimicry. Further, these results suggest that mimicry can dissociate from knowledge of artificiality and experienced emotional unease. Nevertheless, the android mimicry was weaker than spontaneous mimicry of a human, and was evident only in the zygomaticus muscle. Finally, the intentional mimicry of the android was very robust, albeit also weaker than of the human. All of this suggests that the android could, in principle, elicit robust spontaneous mimicry, given the right conditions, as explored next. We will return to the larger theoretical implications of these results for different mimicry theories in the general discussion.

## Study 2

In study 1 we found that people, under some limited conditions, will spontaneously mimic a hyper-realistic android. We proposed that the critical variables relate to individuals' general sense of relation to the android, and not to their emotional comfort, or their belief in its intentionality. If so, one would expect more robust android mimicry in a situation where participants personally encounter the robot. Specifically, as discussed earlier, previous research suggests that direct experience – the robot's actual physical presence – may enhance mimicry. One reason is that direct presence enhances the salience of the android's realistic visual appearance, possibly increasing the sense of human-likeness. In other words, when actually confronted with the android, the salience of humanlike visual features may dominate over any perception of artificial robotic movement. In contrast, when watching that same robot on a screen, the artificiality in movement may be more pronounced (humanlike features appearing less salient on video), leading to weaker mimicry reactions. Interestingly, the same change in salience of detectable human features might also lead to more extreme negative reactions, amplifying the conflict between human-likeness and artificiality. Einstein, the android used in these studies, is actually a disembodied head and shoulders, placed on a platform (see Figure 1A). Although his features are very humanlike, the fact that he is a disembodied figure might cause more discomfort. Thus, testing reactions to the android in face-to-face setting can address questions regarding the relative impact of human-likeness and emotional unease on spontaneous mimicry. In addition, using a real “live” robot opens opportunities to measure mimicry in more methodologically sophisticated ways, such as synchrony between robot facial servos and human facial muscles. For all these reasons, we ran two pretests assessing people's impressions of a physically present android, and, in the separate main experiment, tested people's facial mimicry of a physically present android.

## Methods

### 

#### Pretest 1 – free descriptions of the android

This pretest measured people's spontaneous impressions of the android in a face-to-face interaction. We told 36 separate participants (11 female) that they would be interacting with an android named Einstein, after which they would answer some questions about him. They were placed in a chair facing the robot and asked to freely describe “the android in front of you.” Frequency analysis of these descriptions revealed that participants found the android highly realistic and humanlike. Specifically, 51% used exact words or close synonyms of “realistic”, “real”, “human-like”, “life-like”, and “person.” Spontaneous descriptions for 51% of the participants also included an emotional reaction. As expected, most of these reactions (78%) imply unease about the robot and include words (or close synonyms) of “scary”, “creepy”, or “weird.”

#### Pretest 2 – ratings of the android

This pretest assessed people's general beliefs about intentionality and technology, and their perception of the android's mental states after a face-to-face interaction. Participants were a separate group of 203 undergraduates (148 female). The participants first, and in a separate room, completed the general IDAQ questionnaire [40]. Next, they were sat in a chair facing the android where they examined him making two facial expressions: a happy expression and an angry expression. Participants were then asked to answer the same questions used in Study 1 (i.e., how humanlike is the android, how comfortable do they feel, how aroused/excited do they feel, how sad/bad do they feel, how happy/good do they feel, and how creepy/scary is the android), along with additional questions regarding his mental states. Gender differences in these ratings are reported in File S1.

Analyses on the android-related items showed that it was rated as above moderately humanlike (t-test comparing mean to the midpoint, 5, signifying moderately humanlike: *M* = 5.74, *t*(159) = 4.49, *p*<0.0001, *d* = 0.71), but also creepy (*M* = 6.76, *t*(159) = 10.65, *p*<0.0001, *d* = 1.69). Note, however, that intentionality scores for the android were particularly low (*M* = 1.05, *s* = 1.55), suggesting that although it *appears* human-like, participants do not endow the android with human mental states. Individual differences in intentionality ratings of the android were predicted by individual differences in general intentionality scores (*b* = 0.55, *t*(157) = 7.3, *p*<0.0001) and specific technology-related intentionality questions (*b* = 0.44, *t*(157) = 9.45, *p*<0.0001). As in the previous study, these results suggest that an individual difference in anthropomorphisation of technology in general may lead to anthropomorphisation of the specific android.

**Figure 4:**
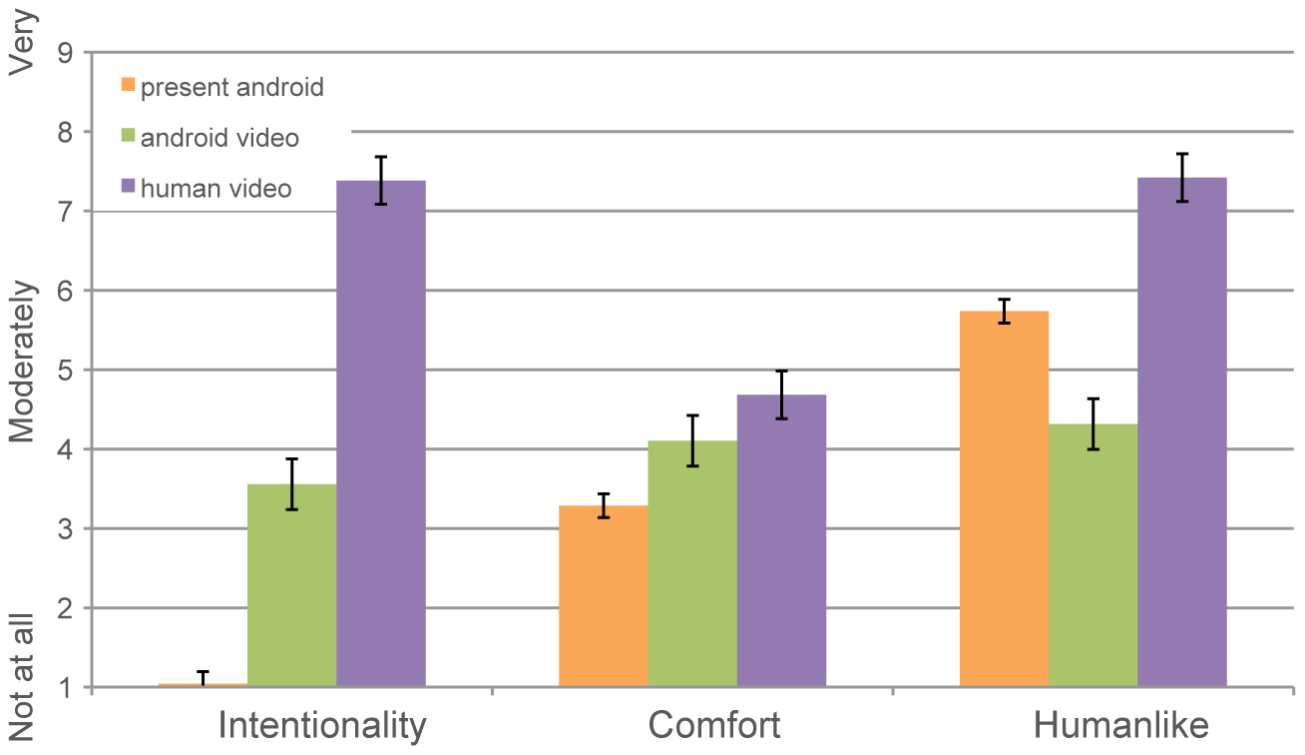
Comparison of ratings for the present android as a robot, on video, and human control. Participants rate the present android as significantly lower in intentionality than his video counterpart, and the human control. Participants feel the least comfortable with the physically present android, yet rate him more humanlike than his likeness in the video. Note: ratings for comfort and humanlike were made on a 1-9 scale, while those on intentionality were made on a 0-10 scale in accordance with the IDAQ [40].

In addition, we compared ratings of the directly present android to those of his videos from Study 1. This allows us to examine whether the physically present android appears more humanlike than the videos of the same android (Figure 4). Interestingly, the “live” android was found to be even less anthropomorphic (using the aforementioned IDAQ questionnaire [40]) than his video counterpart (*M*_present_ = 1.045, *M*_video_ = 3.56, *t*(536.15) = 12.17, *p*<0.0001, *d* = 1.05), and participants were less comfortable viewing him (*M*_present_ = 3.28, *M*_video_ = 4.11, *t*(53.61) = 2.31, *p* = 0.02, *d* = 0.63). However, they found the “live” android significantly more humanlike (*M*_present_ = 5.74, *M*_video_ = 4.32, *t*(56.14) = −3.81, *p* = 0.0004, *d* = 1.02), although still less humanlike than the control (*M*_present_ = 5.74, *M*_control_ = 7.42, *t*(61.94) = 4.97, *p*<0.0001, *d* = 1.26). This suggests that interacting face-to-face with the android leads to heightened impressions of human-likeness, albeit with decreased attributions of internal human-like states – an interesting juxtaposition to which we come back in the general discussion.

#### Participants in the main mimicry experiment

Participants were 23 University of California, San Diego undergraduates (9 female and 14 male). The research protocol was reviewed and approved by the University of California, San Diego Institutional Review Board and all subjects provided written informed consent. Four subjects were excluded due to corrupt data.

#### Robot servo measurement

As mentioned in Study 1, the android was programmed to make human facial expressions using 31 different servos. Of these, several were responsible for making happy and angry faces, which were the expressions used in these mimicry experiments. Figure 1D highlights the relevant servos and the tested links between android and human facial movement. To enable quantitative measurements of synchrony between the android's and a human's facial expressions, we calculated the electrical input to the critical ‘brow’ servo and the critical ‘cheek’ servo (see Figure 1A/D and File S1).

#### Procedure

First, participants were explicitly informed that Einstein is a robot. Mimicry was measured using the same paradigm as in Study 1 (see also File S1). Participants watched the android produce a randomized sequence of angry and happy expressions. This occurred under two conditions: spontaneous mimicry (first) and intentional mimicry (second), each containing 30 expressions. Note that this paradigm allows not only detection of spontaneous mimicry but also the comparison of human-android synchronization under spontaneous and intentional conditions [5], [6]. Mimicry was again measured with facial EMG on the zygomaticus major (cheek muscle) and corrugator supercilii (brow muscle), similar to Study 1.

### Results

We predicted that in this experiment, using a face-to-face setting, participants would spontaneously mimic the android. Again, we predicted that the EMG response generated in the intentional mimicry condition would be stronger than the spontaneous EMG reaction. In addition, mimicry should be evident in the synchronization of participants' EMG signal to the shape and time-course of the android's movements.

**Figure 5:**
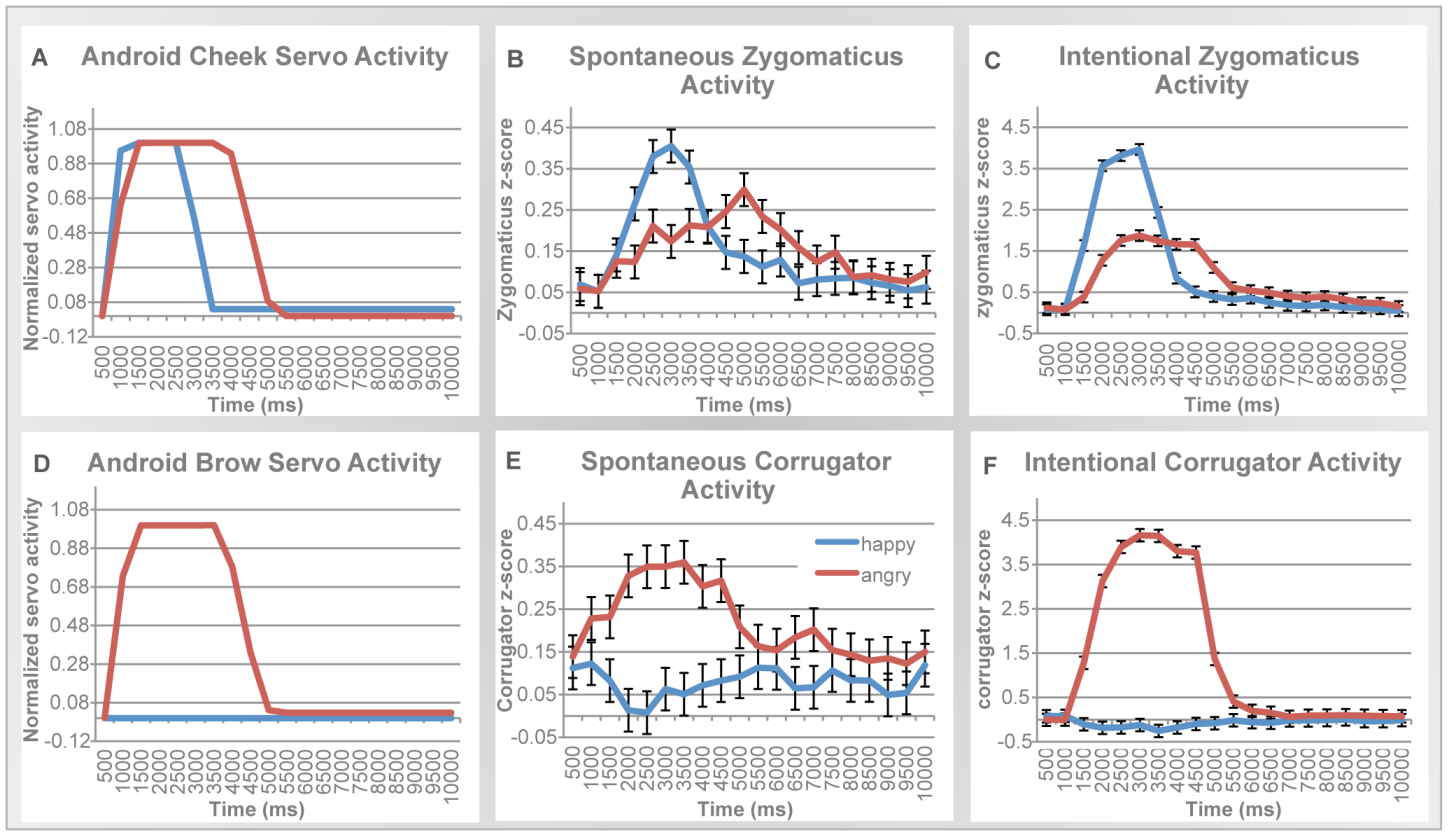
Evidence of facial mimicry in both spontaneous and intentional conditions. Android servo activity (left) compared to spontaneous EMG activity (middle), and intentional EMG activity (right) across the 10 seconds of a trial (note that differences in scale of EMG activity across the y-axis reflect an order of magnitude difference in actual response, whereas the android's servo activity is normalized to peak at the arbitrary value of 1 for comparison). (**A–C**): Cheek and zygomaticus activity. (**D–F**): Brow and corrugator activity. (**A**) Normalized voltage activity sent to the android's ‘cheek’ servo across happy and angry trials. (**B**) Mean spontaneous zygomaticus activity compared across happy and angry trials. (**C**) Mean intentional zygomaticus activity compared across happy and angry trials. (**D**) Normalized voltage activity sent to the android's ‘brow’ servo across happy and angry trials. (**E**) Mean spontaneous corrugator activity compared across happy and angry trials. (**F**) Mean intentional corrugator activity compared across happy and angry trials.

Participants' EMG responses to the robot's facial expressions were analyzed using a repeated-measures MANOVA over all time points in the trial (measured in 500 ms intervals) within each condition (spontaneous mimicry and intentional mimicry). In addition, to examine the nature of very early responses, we conducted repeated-measures MANOVAs in the 1000–2000 window of the trial, with 200 millisecond intervals. The 1000–2000 ms interval was chosen as opposed to the first second because MANOVAs across the full time period showed that participants do not start responding until after 1000 ms, in both conditions – see Figure 5. Again Time was included as a factor in all analyses, so that we could examine and compare the time course of the human expressions with those of the android. Preliminary analyses showed that Gender did not demonstrate any effect on EMG activity in either muscle, and is therefore excluded from results below. Again, recall, and note in Figure 5A, that the android produces cheek activity when making both happy and angry expressions and that this cheek activity occurs earlier for happy than for angry expressions. Correspondingly, participants' zygomaticus EMG activity should increase to both happy and angry expressions, with that increase occurring earlier for happy expressions than angry expressions.

#### Mimicry in the zygomaticus muscle

Figure 5B plots zygomaticus activity in the spontaneous condition and shows clear evidence for spontaneous mimicry. Specifically, zygomaticus activity varied as a function of the robot's expression and time-course, as reflected in a significant Emotion × Time interaction, *F*(1,19) = 2.91, *p*<0.001, *partial η* ^2^ = 0.14. Importantly, spontaneous responses in the zygomaticus to happy expressions significantly increased from the beginning of the trial to the peak at 3.5 seconds, *t*(18) = 2.23, *p* = 0.01, *d* = 0.69. For angry faces, zygomaticus activation rose more gradually until it peaked around 5 s, with a significant difference from the beginning of the trial, *t*(18) = 2.72, *p* = 0.01, *d* = 0.70. These mimicry reactions start rather rapidly, as evidenced in analyses of early responses. A repeated measures MANOVA over the 1000–2000 ms window revealed a marginally significant Emotion × Time interaction, *F*(4,72) = 2.28, *p* = 0.07, *partial η* ^2^ = 0.11. In short, participants' spontaneous zygomaticus reactions closely matched the timing of the robot's cheek movements (see also File S1).

As a control, similar analyses were run on the intentional condition (Figure 5C). Again, humans mimicked: The Emotion × Time interaction was significant (*F*(1,19) = 23.02, *p*<0.001, *partial η* ^2^ = 0.61), and temporal comparisons exhibited similar results to that of the spontaneous condition (see also File S1). Specifically, zygomaticus activation to happy faces rose early and peaked at 3 s, significantly increasing from the beginning of the trial, *t*(18) = 8.43, *p*<0.001, *d* = 2.91. Zygomaticus activation to anger increased later but also peaked at 3 s, *t*(18) = 8.94, *p*<0.001, *d* = 2.57. Further evidence for the rapid initiation of these responses is provided by analyses of early responses, in the 1000–2000 ms window, where there is a main effect of Emotion, *F*(1,19) = 6.27, *p* = 0.02, *partial η* ^2^ = 0.26. Finally, the overall zygomaticus response was stronger in the intentional than spontaneous condition, *F*(1,15) = 17.33, *p* = 0.001, *partial η* ^2^ = 0.54.

#### Mimicry in the corrugator muscle

Next, we focused our analyses on participants' corrugator responses to the robot's ‘brow’ actions. Figure 5E plots corrugator activity in the spontaneous condition. Again, we found evidence for spontaneous mimicry: Corrugator activity was higher overall in response to angry faces compared to happy faces, *F*(1,18) = 5.66, *p* = 0.03, *partial η* ^2^ = 0.24. Once again there was an Emotion × Time interaction, *F*(1,19) = 7.32, *p* = 0.01, *partial η* ^2^ = 0.10. As Figure 5E shows, corrugator activity in response to angry versus happy expressions was greater in the early portion of the trial, peaking at around 2 to 3 seconds, and diminishing later in the trial. Analyses of early (1000–2000 ms interval) responses suggest that these mimicry reactions begin quickly, as revealed in a marginally significant effect of Emotion, *F*(1,19) = 3.46, *p* = 0.08, *partial η* ^2^ = 0.16.

As a control, we performed the same analyses in the intentional condition. As shown in Figure 5F, humans mimicked the android. Specifically, angry expressions elicited higher overall corrugator activity than happy expressions, *F*(1,16) = 111.85, *p*< 0.001, *partial η* ^2^ = 0.88. Once again, mimicry appeared early in the trial, resulting in an Emotion × Time interaction, *F*(1,19) = 67.74, *p*<0.001, *partial η* ^2^ = 0.81. Analyses focusing selectively on early responses confirm this. In the 1000–2000 ms interval there was both a significant main effect of Emotion (*F*(1,19) = 53.12, *p*<0.001, *partial η* ^2^ = 0.75) and significant Emotion × Time interaction (*F*(1,19) = 6.62, *p*<0.001, *partial η* ^2^ = 0.27. Finally, the overall corrugator response was stronger in the intentional than spontaneous condition, *F*(1,16) = 12.55, *p* = 0.003, *partial η* ^2^ = 0.44.

#### Synchronization analyses

Subsequent analyses tested whether participants synchronized their facial responses with the android. We did this by linking the robot's servo voltage to the participants' muscle voltage. More specifically, we compared the electrical input to the android's ‘brow’ and ‘cheek’ servos with participant EMG activity across time.

**Figure 6:**
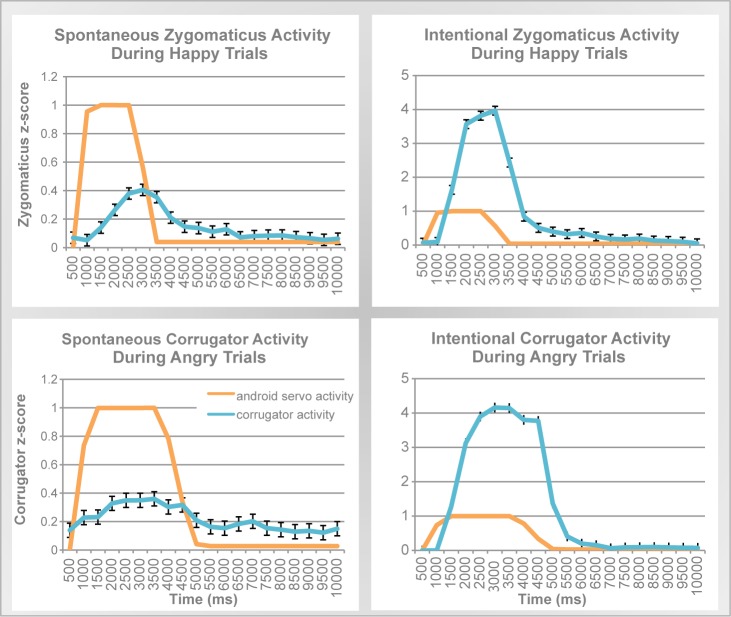
Spontaneous and intentional EMG activity in synchrony with robot servo activity. **TOP**: Robot servo activation for smile movement compared to EMG activity for zygomaticus in spontaneous (left) and intentional (right) mimicry. **BOTTOM**: Robot servo activation for brow lowering compared to EMG activity for corrugator in spontaneous (left) and intentional (right) mimicry. As in Figure 5, note that differences in scale of EMG activity across the y-axis reflect an order of magnitude difference in actual response, whereas the robot servo activity is normalized to peak at the arbitrary value of 1 for comparison.

Figure 6 illustrates the parallels between the action of the robot's servos and the participants' EMG responses. For quantitative comparisons, we computed two measures of synchrony: correlation across time and similarity in response duration.

#### Cross-correlation analyses

A coupling between the leading signal (android) and a trailing signal (human) can be measured with a lagged cross-correlation [43]. This method determines a lag at which maximal correlation occurs between the android's servo activation and time-delayed, congruent human EMG activity (‘brow’ servo and corrugator, and ‘cheek’ servo and zygomaticus) for each trial type (happy and angry). For most participants in the spontaneous condition, such cross-correlations were significant. Notably, when viewing “happy” faces, zygomaticus activity of nearly all subjects (95%) was correlated with cheek servo activity, suggesting that spontaneous cheek mimicry occurs almost universally. Similarly, when viewing “angry” faces, 58% of subjects showed correlations for brow-servo and corrugator activity. The correlations computed across subjects were also significant: cheek-zygo-happy: *r* = 0.52, *p* = 0.01, brow-corr-anger: *r* = 0.41, *p* = 0.05. As expected, participants' intentional mimicry also revealed significant correlations: cheek-zygo-happy: *r* = 0.70, *p*<0.001, brow-corr-anger: *r* = 0.82, *p*<0.001. The timing lags for spontaneous correlations were in the range observed in comparable paradigms exploring human-human facial mimicry, *M* = 1.18 s, *SD* = 2.22 s [5], [6], [44] (see also File S1).

As a control for spurious correlation discovery, we computed EMG-servo correlations for combinations that should not produce synchronization. Specifically, we correlated spontaneous corrugator activity during the happy condition, with servo activation during other conditions (brow servo during angry condition and cheek servo during happy condition). No overall significant correlations were found for any of these combinations. In addition, we computed a planned contrast comparing correlations for the relevant experimental combinations and the control combinations across the emotion and intentional conditions. We found that servo-EMG correlations in the relevant experimental conditions were significantly higher than control (*F*(1,18) = 9.92, *p* = 0.006). Overall, these findings demonstrate synchrony between the robotic facial expressions and the resulting human facial responses.

#### Duration analyses

As an additional measure of synchronization, we examined whether people's facial responses had a similar duration to the android's facial movement. Duration was measured as the full width at half maximum, which computes the width of a signal spike at half of the peak magnitude [43]. We first established that the robot produces movements of differing durations for the different expressions. Indeed, the robot's ‘frown’ servo activity during anger trials (width = 3.48 s) was longer than his ‘smile’ servo activity during happy trials (width = 2.3 s). Given this difference in the android's leading signal (servo activity), we tested whether a similar difference holds for the participants' trailing signal (i.e., EMG response). A repeated measures MANOVA with Expression (corrugator activity for angry, zygomaticus activity for happy) and Condition (spontaneous/intentional) showed that the duration of the participants' response differed as a function of Expression (*F*(1,18) = 14.8, *p* = 0.001, *partial η* ^2^ = 0.45) across both conditions (no main effect or interaction of Condition). Critically, this difference was in the same direction as that of the robot, with longer corrugator activity during angry trials (width = 2.74 s) than zygomaticus activity during happy trials (width = 1.67 s). In short, these data give further support the notion that participants synchronized their facial expressions with the android.

### Discussion

Study 2 explored mimicry responses to the physically present android. Remarkably, almost all participants demonstrated spontaneous mimicry. This is in stark contrast to Study 1 in which spontaneous mimicry only occurred for participants who found the android humanlike (and, even for that subset of participants, the mimicry was weak). Interestingly, the robust spontaneous mimicry in Study 2 occurred despite the fact that pretest participants were less likely to attribute mental states to the physically present android than his video counterpart, were less comfortable with him, and found him more creepy. However, those participants also found the physically present android significantly more humanlike. Thus, it appears that face-to-face contact with the android makes his realistic human appearance highly salient, which in turn facilitates basic processes of mimicry. This suggests that spontaneous mimicry may rely on basic visual cues of humanlike features (and is not dependent on anthropomorphic beliefs).

## General Discussion

We reported two studies exploring whether people engage in mimicry of an android and the conditions that facilitate such mimicry. Overall, these studies clearly show that humans can spontaneously respond to emotional expressions of a hyper-realistic android in a mimicry-like manner: smiling to a smile, frowning to a frown. However, such mimicry depends on several important factors. When such an android appears in a video, mimicry is weak and occurs only for participants who believe the android to be humanlike. However, when such an android is physically present, spontaneous mimicry robustly occurs across virtually all participants. Critically, the physically present android is rated as more humanlike than his video counterpart, suggesting an important role of such perceptions in mimicry. Interestingly, our studies also suggest that mimicry does not depend on attributions of intentional mental capacities to the android. Participants attributed low intentionality to the android in the video, but these ratings were even lower for the physically present android, which participants actually mimicked. Further, in both studies, variation in participants' beliefs in the android's intentionality was not related to mimicry. Even more intriguing is that in both studies spontaneous facial mimicry occurred despite people's emotional unease in response to the android. In fact, the android in face-to-face interaction made participants even more uncomfortable than the android on the video, yet it was in the face-to-face interaction where the android triggered the most robust mimicry.

What are the implications of our findings for theories of mimicry and facial responding? We suggest that our findings fit best with perspectives that view spontaneous mimicry as driven by relatively rudimentary, automatic processes, which are nevertheless modified by some higher-order variables. As mentioned in the introduction, perspectives that emphasize automatic processes include the sensorimotor account [17], the automatic embodiment account [18], as well as the affect-matching account [20]. The current experiments were not designed to differentiate amongst these specific accounts, but a few remarks about the fits of our data to these theories seem appropriate. From the perspective of sensorimotor theories, it is interesting that we observed more robust mimicry when the android was physically present, rather than when it appeared on the computer screen (recall that robust mimicry to humans was observed in this condition). This suggests that mere similarity of an android's face to the human face is not enough to trigger mimicry. This is challenging if one were to see imitation as entirely dependent on low-level sensorimotor processes [17]. Such qualification of pure sensorimotor theories is also suggested by the finding that android mimicry depended on perception of “human-likeness”, which suggests that higher-order processes may play some role. In fact, the most recent formulation of associative sequence-learning (ASL) accounts of mimicry explicitly acknowledges the role of such high-order variables [45].

From the perspective of “affect-matching” theories, it is interesting that the dynamic and shape of peoples' responses so closely matched the actual dynamics and shape of the android's expression, especially in the physically-present condition (Study 2). This includes even aspects of the expression that were not compatible with the notion of induced emotional state (i.e., matching zygomaticus response to observed grimacing in anger). While an affective interpretation of this is open (subjects could be matching “grimacing” states), it seems more plausible to interpret facial behavior of our participants as reflecting more motor-driven, imitative processes. Future studies with measures of underlying affective state may address this issue.

One conclusion that can be made firmly is that our findings cannot be easily accommodated by theories that explain mimicry as a form of strategic simulation aimed at understanding another agent's intentional states from within. Clearly, our data show that mimicry occurs even when people clearly do not believe that the other agent is conscious, has free will, or experiences emotions – the hallmarks of an intentional being. Of course, this does not challenge the viability of strategic simulation theories for explaining how humans understand other humans. It is also possible to see mimicry of an android as a kind of overgeneralization of a simulation process that works well in a typical social life.

Furthermore, our findings challenge theories that are strongly committed to the idea that mimicry processes require positive emotion. Clearly, in our case a robot triggered mimicry, even when its appearance made people uncomfortable and was judged to be creepy, suggesting that our android may fall into the uncanny valley. Again, our findings do not challenge earlier reports that human imitation and empathy strongly depends on initial liking [19], [25], [37], [38]. However, they do suggest that such reports obtained in a human-to-human context may not generalize to human-android interactions.

Finally, it is worth highlighting some implications of our results for human-robot interactions. Most importantly, the reported evidence of spontaneous facial mimicry to an artificial agent reveals a capacity for a rudimentary connection between humans and androids. As such, future robot developers could use mimicry as a real-time feedback signal to create more meaningful human-robot interactions. Further, it appears that people's experiences interacting with physically-present androids are qualitatively different than interactions with virtual agents on a screen. This calls for further research on the role of agents' actual physical presence. Lastly, we believe that the present work highlights how investigating basic psychological processes with artificial agents can teach us, humans, about ourselves, but also reveals the potential effects of incorporating such agents in an increasingly technologically complex and sophisticated world.

## Supporting Information

File S1 **Supplementary Methods and Analyses.**
(DOCX)

## References

[1] Preston SD, De Waal FB (2002) Empathy: Its ultimate and proximate bases. Behavioral and Brain Sciences 25: 1–20. 12625087 10.1017/s0140525x02000018

[2] Tomasello M (1999) The human adaptation for culture. Annual Review of Anthropology 28: 509–529.

[3] Hatfield E, Cacioppo JT, Rapson RL (1992) Primitive emotional contagion. In: Clark MS, editor. Emotion and Social Behavior. Review of personality and social psychology. Thousand Oaks, CA, US: Sage Publications, Vol. 14. pp. 151–177.

[4] Brothers L (1990) The neural basis of primate social communication. Motiv Emot 14: 81–91 10.1007/BF00991637

[5] Dimberg U (1982) Facial Reactions to Facial Expressions. Psychophysiology 19: 643–647 10.1111/j.1469-8986.1982.tb02516.x 7178381

[6] McIntosh DN, Reichmann Decker A, Winkielman P, Wilbarger JL (2006) When the social mirror breaks: deficits in automatic, but not voluntary, mimicry of emotional facial expressions in autism. Developmental Science 9: 295–302. 16669800 10.1111/j.1467-7687.2006.00492.x

[7] Oberman LM, Winkielman P, Ramachandran VS (2007) Face to face: blocking facial mimicry can selectively impair recognition of emotional expressions. Soc Neurosci 2: 167–178 10.1080/17470910701391943 18633815

[8] Stel M, Van Knippenberg A (2008) The Role of Facial Mimicry in the Recognition of Affect. Psychological Science 19: 984–985 10.1111/j.1467-9280.2008.02188.x 19000207

[9] Carr L, Iacoboni M, Dubeau M-C, Mazziotta JC, Lenzi GL (2003) Neural mechanisms of empathy in humans: A relay from neural systems for imitation to limbic areas. Proceedings of the National Academy of Sciences 100: 5497–5502 10.1073/pnas.0935845100 PMC154373 12682281

[10] Chartrand TL, Bargh JA (1999) The chameleon effect: the perception-behavior link and social interaction. J Pers Soc Psychol 76: 893–910. 10402679 10.1037//0022-3514.76.6.893

[11] Hasson U, Ghazanfar AA, Galantucci B, Garrod S, Keysers C (2012) Brain-to-brain coupling: a mechanism for creating and sharing a social world. Trends in Cognitive Sciences 16: 114–121 10.1016/j.tics.2011.12.007 22221820 PMC3269540

[12] Winkielman P, Kavanagh L (2013) The embodied perspective on emotion-cognition interactions. In: Robinson MD, Watkins ER, Harmon-Jones E, editors. Handbook of cognition and emotion. New York NY: Guilford Press. pp. 212–230.

[13] Gratch J, Okhmatovskaia A, Lamothe F, Marsella S, Morales M, et al. (2006) Virtual Rapport. In: Gratch J, Young M, Aylett R, Ballin D, Olivier P, editors. Intelligent Virtual Agents. Lecture Notes in Computer Science. Springer Berlin Heidelberg. pp. 14–27. Available: http://link.springer.com/chapter/10.1007/11821830_2. Accessed 2013 February22.

[14] Riek LD, Paul PC, Robinson P (2010) When my robot smiles at me: Enabling human-robot rapport via real-time head gesture mimicry. J Multimodal User Interfaces 3: 99–108 10.1007/s12193-009-0028-2

[15] Coradeschi S, Ishiguro H, Asada M, Shapiro SC, Thielscher M, et al (2006) Human-Inspired Robots. IEEE Intell Syst 21: 74–85 10.1109/MIS.2006.72

[16] Tanaka F, Cicourel A, Movellan JR (2007) Socialization between toddlers and robots at an early childhood education center. Proceedings of the National Academy of Sciences 104: 17954–17958 10.1073/pnas.0707769104 PMC2084278 17984068

[17] Heyes C (2011) Automatic imitation. Psychol Bull 137: 463–483 10.1037/a0022288 21280938

[18] Press C, Bird G, Flach R, Heyes C (2005) Robotic movement elicits automatic imitation. Cognitive Brain Research 25: 632–640 10.1016/j.cogbrainres.2005.08.020 16344220

[19] Niedenthal PM, Mermillod M, Maringer M, Hess U (2010) The Simulation of Smiles (SIMS) Model: Embodied Simulation and the Meaning of Facial Expression. Behavioral and Brain Sciences 33: 417–433 10.1017/S0140525X10000865 21211115

[20] Dimberg U, Thunberg M, Elmehed K (2000) Unconscious facial reactions to emotional facial expressions. Psychological Science 11: 86. 11228851 10.1111/1467-9280.00221

[21] Moody EJ, McIntosh DN, Mann LJ, Weisser KR (2007) More than mere mimicry? The influence of emotion on rapid facial reactions to faces. Emotion 7: 447–457 10.1037/1528-3542.7.2.447 17516821

[22] Goldman AI (2006) Simulating minds: the philosophy, psychology, and neuroscience of mindreading. Oxford University Press. 378 p.

[23] Chouchourelou A, Golden A, Shiffrar M (2013) What Does “Biological Motion” really mean? Differentiating visual percepts of human, animal, and non-biological motions. In: Johnson K, Shiffrar M, editors. People Watching: Social, Perceptual, and Neurophysiological Studies of Body Perception. Oxford Series in Visual Cognition. New York NY: Oxford University Press. pp. 63–81.

[24] Nadel J, Simon M, Canet P, Soussignan R, Blancard P, et al.. (2006) Human responses to an expressive robot. Proceedngs of the sixth international workshop on epigentic robotics. Lund University Cognitive Studies, Vol. 128. pp. 79–86.

[25] Likowski KU, Mühlberger A, Seibt B, Pauli P, Weyers P (2008) Modulation of facial mimicry by attitudes. Journal of Experimental Social Psychology 44: 1065–1072 10.1016/j.jesp.2007.10.007

[26] Weyers P, Mühlberger A, Hefele C, Pauli P (2006) Electromyographic responses to static and dynamic avatar emotional facial expressions. Psychophysiology 43: 450–453 10.1111/j.1469-8986.2006.00451.x 16965606

[27] Weyers P, Mühlberger A, Kund A, Hess U, Pauli P (2009) Modulation of facial reactions to avatar emotional faces by nonconscious competition priming. Psychophysiology 46: 328–335 10.1111/j.1469-8986.2008.00771.x 19207205

[28] Kiesler S, Powers A, Fussell SR, Torrey C (2008) Anthropomorphic Interactions with a Robot and Robot–like Agent. Social Cognition 26: 169–181 10.1521/soco.2008.26.2.169

[29] Trope Y, Liberman N (2010) Construal-level theory of psychological distance. Psychological Review 117: 440–463 10.1037/a0018963 20438233 PMC3152826

[30] Sanchez-Vives MV, Slater M (2005) From presence to consciousness through virtual reality. Nature Reviews Neuroscience 6: 332–339 10.1038/nrn1651 15803164

[31] Goldman AI, Sripada CS (2005) Simulationist Models of Face-Based Emotion Recognition. Cognition 94: 193–213. 15617671 10.1016/j.cognition.2004.01.005

[32] Epley N, Waytz A, Cacioppo JT (2007) On Seeing Human: A Three-Factor Theory of Anthropomorphism. Psychological Review 114: 864–886. 17907867 10.1037/0033-295X.114.4.864

[33] Waytz A, Gray K, Epley N, Wegner DM (2010) Causes and consequences of mind perception. Trends in Cognitive Sciences 14: 383–388 10.1016/j.tics.2010.05.006 20579932

[34] Freud S (1919) Das Unheimliche. Gesammelte Werke 12: 229–268.

[35] Mori M (1970) The uncanny valley. Energy 7: 33–35.

[36] Saygin AP, Chaminade T, Ishiguro H, Driver J, Frith C (2012) The thing that should not be: predictive coding and the uncanny valley in perceiving human and humanoid robot actions. Soc Cogn Affect Neurosci 7: 413–422 10.1093/scan/nsr025 21515639 PMC3324571

[37] Bourgeois P, Hess U (2008) The impact of social context on mimicry. Biological Psychology 77: 343–352 10.1016/j.biopsycho.2007.11.008 18164534

[38] Lakin JL, Jefferis VE, Cheng CM, Chartrand TL (2003) The chameleon effect as social glue: Evidence for the evolutionary significance of nonconscious mimicry. Journal of nonverbal behavior 27: 145–162.

[39] Wu T, Butko NJ, Ruvulo P, Bartlett MS, Movellan JR (2009) Learning to Make Facial Expressions. Proceedings of the 2009 IEEE 8th International Conference on Development and Learning. IEEE. pp. 1–6.

[40] Waytz A, Cacioppo J, Epley N (2010) Who sees human? The stability and importance of individual differences in anthropomorphism. Perspectives on Psychological Science 5: 219–232 10.1177/1745691610369336 24839457 PMC4021380

[41] Fridlund AJ, Cacioppo JT (1986) Guidelines for human electromyographic research. Psychophysiology 23: 567–589. 3809364 10.1111/j.1469-8986.1986.tb00676.x

[42] Tassinary LG, Cacioppo JT (2000) The skeletomotor system: Surface electromyography. Handbook of psychophysiology 2: 163–199.

[43] Orfanidis SJ (1988) Optimum Signal Processing: An Introduction. 2nd ed. New York, NY: Macmillan.

[44] Davila Ross M, Menzler S, Zimmermann E (2008) Rapid facial mimicry in orangutan play. Biology letters 4: 27. 18077238 10.1098/rsbl.2007.0535 PMC2412946

[45] Cook R, Bird G, Catmur C, Press C, Heyes C (2014) Mirror neurons: From origin to function. Behavioral and Brain Sciences 37: 177–192 10.1017/S0140525X13000903 24775147

