# Supplementary material for: Bridging the Mechanical and the Human Mind: Spontaneous Mimicry of a Physically Present Android
Source: PLoS One. 2014 Jul 18;9(7):e99934. doi: 10.1371/journal.pone.0099934 (PMC4103778; doi:10.1371/journal.pone.0099934)
Supplement: File S1 — Supplementary Methods and Analyses. (DOCX) [file pone.0099934.s001.docx]

Supplementary Material for

Bridging the Mechanical and the Human Mind: Spontaneous Mimicry of a Physically Present Android

Galit Hofree, Paul Ruvolo, Marian Stewart Bartlett and Piotr Winkielman

**Supplementary Methods**

**Robot Facial Expression Design**

Certified experts in the study of human facial expressions choreographed the movement of each of Einstein’s 31 servos in order to create a set of expressions that matched the expressions of basic emotion as described in Ekman and Friesen’s FACS manual [1]. A short video of Einstein displaying both emotions can be found on this page: [http://pages.ucsd.edu/~pwinkielman/einstein_happy_angry.mov](http://pages.ucsd.edu/~pwinkielman/einstein_happy_angry.mov)

While the servos were situated in order to simulate human facial muscles as closely as possible, due to the differences between human tissue and Einstein’s synthetic materials, multiple servos were often required to simulate the facial surface movement caused by a particular facial muscle. Twenty one servomotors were used in the production of Einstein’s happy face that lasted a total of 3 seconds from onset to offset. 23 servomotors were used in the production of Einstein’s anger expression that lasted a total of 4.5 seconds from onset to offset. 5 servos participated in specifically moving the brow during an angry expression (movement which corresponds to corrugator activity in the human expression of angry), and 4 servos move the lip corner during a happy expression (corresponding to zygomaticus activity during a happy expression). Each set of servos share a basic pattern of activation (onset, offset and duration of activity). One of each of these servo sets was chosen as a representative ‘cheek’ and ‘brow’ servo for comparisons with between robot ‘cheek’ and ‘brow’ activity, and human zygomaticus and corrugator activity.

**Study 2 Procedure**

Experiment code was written in Java v6. The experiment included four phases: practice, automatic mimicry, intentional mimicry and emotion recognition. We followed the paradigm used in McIntosh *et al.* [2]. We decided to include a practice phase, in order to enable subjects to become familiar with the robot and his facial expressions. During this phase, subjects viewed all six emotional facial expressions twice, randomly presented. During the spontaneous mimicry phase, subjects were requested to ‘Please sit comfortably and watch what Einstein does.’ During the voluntary mimicry phase they were asked to ‘Please try and make the same face as Einstein.’ Both involved random intermixed presentation of angry and happy facial expressions – fifteen of each expression, on each block. Finally during the recognition phase, subjects were presented with each of the six expressions five times, randomly intermixed. Subjects were requested to press a certain key for each emotion that Einstein expressed. This phase was introduced so that we could be sure participants could recognize Einstein’s expressions. In order to time-lock EMG measurements with the android’s expressions, a signal was simultaneously sent to the EMG acquiring system and to the android’s servos for each expression.

**EMG data processing**

EMG was measured by pairs of 4-mm electrodes over the regions of zygomaticus major (cheek) and corrugator supercilii (brow), according to EMG processing standards [3]. For the zygomaticus major muscle, the first electrode was placed in the middle of an imaginary line between the lip corner at rest, and the point where the jaws meet (approximately near the ear lobe). The second electrode was placed a collar width (approximately 1 cm) posterior to the first. For the corrugator supercilli muscle, the first electrode was placed right above the eyebrow, on an invisible vertical line from the corner of the eye up. The second electrode was placed a collar width posterior to the first (following the eyebrow arch).

AcqKnowledge software (Biopac Systems, Goleta, CA) along with Biopac (Biopac Systems, Goleta, CA) were employed to acquire the EMG signal. The amplified EMG signals were filtered online with a low-pass of 500 Hz and a high-pass of 10 Hz, sampled at a rate of 2000 Hz, and then integrated and rectified using Mindware EMG software, version 2.52 (MindWare Technologies Ltd., Ohio, USA).

**EMG Data Reduction**

**Study 1.** EMG data was analyzed using Matlab (version R2008b, The Mathworks, Natick, MA) JMP (version 8, SAS Institute Inc., Cary, NC) and SPSS (version 17, IBM Corporation, Armonk, NY). Data were first averaged in 500 ms intervals across a trial (i.e. 12 data points for a 6 second trial). Extreme values (values greater than 3 standard deviations away from the mean) were excluded from the analysis. Next, data were standardized within participant and within each muscle. A mean of the activity during time window of 2000 ms before the onset of a video served as a baseline. We calculated baseline-corrected activity for each participant and each muscle across the 6 second trial. Finally, we averaged baseline corrected EMG activity within 500 ms intervals across trials, within individual, muscle, block (spontaneous, intentional), object, video type and condition (happy, angry).

For analyses of rapid mimicry responses, we processed the data separately using 200 ms intervals across the first second of the trial (5 data points). The rest of the processing was conducted similarly to the main dataset.

**Study 2.** EMG data were processed in a similar manner to that of study 1, with a few exceptions. Data were first averaged in 500 ms intervals across a trial (i.e. 20 data points for a 10 second trial). EMG data from the practice block and the recognition block were excluded from the rest of the analyses, due to the amount of noise they introduced when standardizing activity. Extreme values (values greater than 3 standard deviations away from the mean) were excluded from the analysis. Next, data were standardized within participant and within each muscle. A median of the activity during time window of 2000 ms before a command was sent to Einstein (marking the beginning of a trial) served as a baseline. We calculated baseline-corrected activity for each participant and each muscle across the 10 second trial. Finally, we averaged baseline corrected EMG activity within 500 ms intervals across trials, within individual, muscle, block (spontaneous, intentional), and condition (happy, angry).

For analyses of rapid mimicry responses, we processed the data separately using 200 ms intervals across the second second of the trial (5 data points). The rest of the processing was conducted similarly to the main dataset. The second second as opposed to the first second was chosen after MANOVAs across the full time period presented evidence that participants don’t respond until the second second, in both conditions – see Figure 5, main paper.

**Supplementary Statistical Analyses**

**Gender effects**

Overall, we found minimal gender effects in both studies. Those we did find are detailed below. Interestingly, we found that males displayed a stronger deliberate mimicry reaction in both muscles as compared to females (Study 1. However, overall mimicry effects in this condition remained the same whether Gender was included or not.

**Study 1 – Post Experiment Ratings.** Although overall we did not find a difference in negative ratings of the android and the control, when including Gender into the analysis (using a mixed model Agent x Gender MANOVA), we found a significant Gender x Agent interaction (*F*(1,36)=5.20, *p*=0.03, *partial η^2^*=0.085). It appears that females find the android significantly more negative than the human control (*M_android_*=2.67, *M_control_*=3.6, *t*(23)=2.71, *p*=0.01, *d*=1.13), while males do not rate them differently (*M_android_*=3.5, *M_control_*=3, *p*=n.s.).

**Study 1 – Deliberate EMG activity.** Gender effects were analyzed by including them in an omnibus ANOVA with Agent, Emotion and Time as within-subject factors, for each muscle. Males’ mimicry responses are stronger than females in the corrugator muscle during the deliberate condition, as demonstrated by a Gender x Emotion interaction (*F_(_1,27)*=17.69, *p*=0.05, *partial η^2^*=0.14). However, even when including Gender in the analysis, effects of Emotion (*F(1,27)*=180.19, *p*<0.0001, *partial η^2^*=0.87) and Agent x Emotion (*F(1,27)*=9.49, *p*=0.005, *partial η^2^*=0.26) are both highly significant, suggesting that the main effects of interest are not affected by gender differences. In the zygomaticus muscle, the genders differed in their deliberate mimicry reactions to the two agents, as can be seen in both a Gender x Agent x Emotion interaction (*F(1,27)*=4.77, *p*=0.04, *partial η^2^*=0.15), and a Gender x Agent x Emotion x Time interaction (*F(11,297)*=3.807, *p*<0.0001, *partial η^2^*=0.124). Overall, their mimicry reaction differ across agents, as suggested by a Gender x Agent x Time interaction (*F(11,297)*=2.967, *p*=0.001, *partial η^2^*=0.10). Again, the main effects described in the manuscript remain significant. Examining each agent separately reveals that males mimic the control more strongly than the android. This is demonstrated by a Gender x Emotion x Time interaction (*F(11,297)*=4.402, *p*<0.0001, *partial η^2^*=0.14). Overall, it appears that males are more reactive in both muscles during the intentional mimicry phase, but are especially more so to the human control than to the android.

**Study 2 – Ratings of the android.** The genders did not differ on most ratings of the android. However, females rated the android as significantly more creepy (*M_males_*=6.15, *M_females_*=7.01, *t(54)*=-2.02, *p*=0.05, *d*=0.55), and less positive (*M_males_*=4.92, *M_control_*=3.77, *t(75)*=3.25, *p*=0.002, *d*=0.75). This corresponds with the gender differences found in Study 1, in which females found the android more negative.

Analyses of rapid mimicry responses over the first second of the trial

Mimicry reactions can be very quick – initiated around 500 milliseconds from exposure to a stimulus [4,5]. Therefore, in addition to the main analyses of EMG activity over the course of a 6 second trial, we conducted repeated measures MANOVAs in 200 millisecond intervals, over the course of the first second of the trial. These analyses enabled us to examine whether there are particular differences in early responses between the agents.

Study 1 – Rapid intentional mimicry responses. Both the zygomaticus and corrugator demonstrate rapid initiation of mimicry response to both the android and the control. For the android, we find both a main effect of Emotion (*zygomaticus: F*(1,28)=52.29, *p*<0.0001, *partial η*^2^=0.65; *corrugator:* *F*(1,28)=57.44, *p*<0.0001, *partial η*^2^=0.7) and Emotion x Time interaction (*zygomaticus:* *F*(4,112)=34.81, *p*<0.0001, *partial η*^2^=0.55; *corrugator*: *F*(4,112)=26.58, *p*<0.0001, *partial η*^2^=0.49). Both a main effect of Emotion (*zygomaticus:* *F*(1,28)=33.64, *p*<0.0001, *partial η*^2^=0.55; *corrugator*: *F*(1,28)=49.00, *p*<0.0001, *partial η*^2^=0.64) and Emotion x Time (*zygomaticus:* *F*(4,112)=12.63, *p*<0.0001, *partial η*^2^=0.31; *corrugator*: *F*(4,112)=31.70, *p*<0.0001, *partial η*^2^=0.53) can be found in response to the control as well.

When running a MANOVA with Agent as a factor (in addition to Emotion and Time), we again find similar results to those found across the entire trial. For the zygomaticus, there is a significant main effect of Agent (*F*(1,28)=22.43, *p*<0.0001, *partial η*^2^=0.45), as well as a significant Agent x Time interaction (*F*(4,112)=4.97, *p*=0.001, *partial η*^2^=0.15), and Agent x Emotion x Time interaction (*F*(4,112)=3.72, *p*=0.007, *partial η*^2^=0.12), besides the expected effects of Emotion (*F*(1,28)=62.07, *p*<0.0001, *partial η*^2^=0.69) and Emotion x Time (*F*(4,112)=30.34, *p*<0.0001, *partial η*^2^=0.52). For the corrugator, there is a significant main effect of Agent (*F*(1,28)=11.70, *p*=0.002, *partial η*^2^=0.30) and a significant Agent x Time interaction (*F*(4,112)=5.32, *p*=0.001, *partial η*^2^=0.16), besides the expected effects of Emotion (*F*(1,28)=59.49, *p*<0.0001, *partial η*^2^=0.68) and Emotion x Time (*F*(4,112)=35.85, *p*<0.0001, *partial η*^2^=0.56). These all suggest that very early on, participants differentiate between the agents and respond accordingly.

Study 2 - Differences in Time-flow of Zygomaticus Reactions to Happy and Angry Faces

Spontaneous mimicry was detected in the zygomaticus, in both the happy and angry conditions. Critically, these mimicry responses present different time-flow characteristics, matching the differences in the robot’s expressions of angry and happy faces. Einstein’s happy face rises early and has a short duration, whereas his angry expression rises slightly slower and lasts longer (see figure 5A, main paper).

**Time-flow characteristics of spontaneous mimicry.** First, for happy faces, human peak activation happens relatively quickly, around 3.5s, *t*(18) = 2.23, *p* = 0.01, *d* = 0.69, and lasts only until 5s, *t*(18) = -2.09, *p* = 0.03, *d* = 0.50. For angry faces, however, human zygomaticus activation slowly picks up and peaks at 5s, *t*(18) = 2.72, *p* < 0.01, *d* = 0.70, while offset occurs at 7s: *t(*18) = -2.59, *p* = 0.009, *d* = 0.49 (see figure 5B, main paper).

**Time-flow characteristics of intentional mimicry*.*** Similar differences of time course of activity are found in intentional zygomaticus mimicry. Happy face peak activation happens at 3s, *t*(18) = 8.43, *p* < 0.001, *d* = 2.91, and, as in the spontaneous condition, activity offset occurs at 5s, *t*(18) = -1.81, *p* = 0.04, *d* = 2.68. Angry face activation peaks again at 3s: *t*(18) = 8.43, p < 0.001, *p* = 0.04, *d* = 2.56. However, this response lasts longer than in the happy condition, offsetting at 7s, *t*(18) = -6.73, *p* < 0.001, *d* = 2.01 (see figure 5C, main paper).

**Study 2 - Computing cross-correlations as a measure of robot human synchrony**

In order to quantitatively measure how well participants really mimicked the robot, we computed the cross-correlation between each subject and the robot. This method is used in situations in which there is a difference in timing, such that one signal (human participants) lag behind that of the leading signal (Einstein). This method finds the offset, or delay, which generates the largest correlation between the two signals, out of a set of all possible delays. This correlation can then be considered a correlation between Einstein’s servo activity, and the participant’s matching muscle activity. Most of our experimental subjects produced significant correlations between ‘cheek’ servo and zygomaticus for happy trials, and ‘brow’ servo and corrugator, for angry trials (see main paper). Once correlations were calculated for each participant, we computed an overall correlation between servo activity and participants’ spontaneous muscle activation for each type of trial. In order to do this, we first standardized individual correlations using a Fisher z-transformation, and then calculated the significance of the overall correlation. These produced significant overall correlations, as demonstrated in the main paper.

**Correlations between spontaneous and intentional mimicry.** We compared spontaneous mimicking responses to intentional responses, in order to test whether these two conditions were similar to each other. Using the cross-correlation method, we found a significant overall correlation for zygomaticus activity in both happy and angry conditions (happy: *r* = 0.4371, *p* = 0.03; angry: *r* = 0.4173, *p* = 0.04). This suggests that intentional and spontaneous mimicry are similar in shape, at least for zygomaticus reactions to angry and happy expressions.

References

1. Ekman P, Friesen WV (1978) Facial action coding system: A technique for the measurement of facial movement. Consulting Psychologists Press, Palo Alto, CA.

2. McIntosh DN, Reichmann Decker A, Winkielman P, Wilbarger JL (2006) When the social mirror breaks: deficits in automatic, but not voluntary, mimicry of emotional facial expressions in autism. Developmental Science 9: 295–302.

3. Tassinary LG, Cacioppo JT (2000) The skeletomotor system: Surface electromyography. Handbook of psychophysiology 2: 163–199.

4. Dimberg U, Thunberg M, Elmehed K (2000) Unconscious facial reactions to emotional facial expressions. Psychological Science 11: 86.

5. Dimberg U (1982) Facial Reactions to Facial Expressions. Psychophysiology 19: 643–647. doi:10.1111/j.1469-8986.1982.tb02516.x.
